# Exenatide induces aortic vasodilation increasing hydrogen sulphide, carbon monoxide and nitric oxide production

**DOI:** 10.1186/1475-2840-13-69

**Published:** 2014-04-02

**Authors:** Eszter Sélley, Szilárd Kun, István András Szijártó, Boglárka Laczy, Tibor Kovács, Ferenc Fülöp, István Wittmann, Gergő A Molnár

**Affiliations:** 1 2nd Department of Medicine and Nephrological Center, University of Pécs, Hungary, 1. Pacsirta St., H-7624 Pécs, Hungary; 2 Medical Clinic for Nephrology and Internal Intensive Care, Charité Campus Virchow Klinikum and Experimental and Clinical Research Center (ECRC), Max Delbrück Center for Molecular Medicine, Berlin, Germany; 3 Institute of Pharmaceutical Chemistry, University of Szeged, Szeged, Hungary

**Keywords:** Glucagon-like-peptide-1, Exenatide, Vasodilation, Aortic rings, Central blood pressure

## Abstract

**Background:**

It has been reported that GLP-1 agonist exenatide (exendin-4) decreases blood pressure. The dose-dependent vasodilator effect of exendin-4 has previously been demonstrated, although the precise mechanism is not thoroughly described. Here we have aimed to provide *in vitro* evidence for the hypothesis that exenatide may decrease central (aortic) blood pressure involving three gasotransmitters, namely nitric oxide (NO) carbon monoxide (CO), and hydrogen sulphide (H_2_S).

**Methods:**

We determined the vasoactive effect of exenatide on isolated thoracic aortic rings of adult rats. Two millimetre-long vessel segments were placed in a wire myograph and preincubated with inhibitors of the enzymes producing the three gasotransmitters, with inhibitors of reactive oxygen species formation, prostaglandin synthesis, inhibitors of protein kinases, potassium channels or with an inhibitor of the Na^+^/Ca^2+^-exchanger.

**Results:**

Exenatide caused dose-dependent relaxation of rat thoracic aorta, which was evoked via the GLP-1 receptor and was mediated mainly by H_2_S but also by NO and CO. Prostaglandins and superoxide free radical also play a part in the relaxation. Inhibition of soluble guanylyl cyclase significantly diminished vasorelaxation. We found that ATP-sensitive-, voltage-gated- and calcium-activated large-conductance potassium channels are also involved in the vasodilation, but that seemingly the inhibition of the KCNQ-type voltage-gated potassium channels resulted in the most remarkable decrease in the rate of vasorelaxation. Inhibition of the Na^+^/Ca^2+^-exchanger abolished most of the vasodilation.

**Conclusions:**

Exenatide induces vasodilation in rat thoracic aorta with the contribution of all three gasotransmitters. We provide *in vitro* evidence for the potential ability of exenatide to lower central (aortic) blood pressure, which could have relevant clinical importance.

## Background

Exenatide (exendin-4), a glucagon-like-peptide (GLP-1) receptor agonist, binds to the GLP-1 receptor (GLP-1R), which is present as well as elsewhere on endothelial and vascular smooth muscle cells [1,2]. GLP-1R is highly expressed in rat thoracic aorta [3]. Several studies have demonstrated that GLP-1 causes relaxation of arteries in a concentration-dependent manner, however, findings on the putative mechanism of the vasodilator effect of GLP-1 appear controversial [3-7]. Both endogenous GLP-1 and exendin-4 induced vasorelaxation of the rat thoracic aorta via the involvement of ATP-sensitive potassium channels (K_ATP_) and cyclic-adenosine monophosphate (cAMP) [3]. In rat femoral artery, GLP-1 caused endothelium-independent, dose-dependent relaxation [4], while in pulmonary arteries the vasodilation due to GLP-1 was described as endothelium-dependent [5,6]. GLP-1 was also described as elevating plasma nitric oxide (NO) levels, hence being a potent endothelial vasodilator [7]. Besides the activation of K_ATP_ channels, GLP-1 is also involved in modulating the activity of the voltage-gated potassium channels (K_v_) [3,8,9].

NO, carbon monoxide (CO), and hydrogen sulphide (H_2_S) are gaseous signalling molecules, mediating vasodilatory effects in the arterial tree [10]. They are all known to act via the formation of cyclic nucleotides (cAMP/cGMP) and activation of intracellular protein kinases (PKA/PKG) and to cause vasodilation via modulating potassium channels [10]. The vasorelaxation in response to H_2_S often evolves by the activation of K_ATP_ channels and the KCNQ-type voltage gated potassium channels [10,11]. In mice models of hypertension KCNQ channel openers are proven to reduce arterial blood pressure [12]. Both CO and superoxide (O_2_^–•^) are known activators of K_v_ channels [13]. The vasodilator effect of CO partially lies in the activation of these channels; moreover, the activation of PKG or the activation of PKA by NO results in the opening of these channels [10,13].

The aim of this study was to determine the mediators and second messengers involved in the vasodilator effect of exenatide, therefore we studied the effect of exenatide on gasotransmitters and ion channels in rat thoracic aorta.

## Materials and methods

### Animals

We performed our experiments with the permission of the Hungarian Local Animal Experiment Committee in accordance with the ‘Principles of laboratory animal care’ (NIH publication no. 85–23, revised 1985). Adult, 10–12 week old (280–340 g) male CFY Sprague-Dawly rats were kept on a standard chow. Animals were originally purchased from Charles River Laboratories GmbH (Sulzfeld, Germany). On the day of the experiment, after anesthetization with ether, rats were killed by decapitation using guillotine.

### Vasoreactivity experiments

The thoracic aorta was gently excised from rats and was placed in oxygenated (95% O_2_/5% CO_2_), ice-cold Krebs solution (119 mM NaCl, 4.7 mM KCl, 1.2 mM KH_2_PO_4_, 25 mM NaHCO_3_, 1.2 mM Mg_2_SO_4_, 11.1 mM glucose, 1.6 mM CaCl_2_*2H_2_O, pH 7.4). As described earlier, the perivascular fat and connective tissue were gently removed [12,14], and 2 mm long segments of the vessel were mounted on two stainless steel wires (40 μm in diameter), and placed in 5 ml organ baths of a wire myograph (Danish Multimyograph Model 610 M, DMT- USA Inc., Atlanta, GA, USA). Vessel rings were kept in Krebs solution at 37 ºC, pH 7.4 and were continuously oxygenated with a gas containing 95% O_2_ and 5% CO_2_. The rings were placed under a tension of 1 g [3]. Isometric tension was continuously recorded. After a 30 minutes resting period, vessel rings were preconstricted with 100 nM epinephrine as described earlier, which in our previously performed experiments had shown 60% contraction force of the 60 mM KCl contraction [15]. After all vessel segments had reached a stable contraction plateau, increasing doses of exenatide (Byetta® injection, Bristol-Myers Squibb–AstraZeneca, Budapest, Hungary) were administered to the organ baths, and relaxant responses were assessed. The dose of exenatide that was applied to relax the aorta correlated with the dose of epinephrine we used to preconstrict the vessels. Plasma epinephrine level is approximately 30 pM at rest, while in our experiments we used 100 nM, which is a 3000 times higher concentration [16]. The plasma exenatide level was found to be 70 pM, while in our experiments we used a 4500 times higher concentration [17].

In order to identify the extracellular and intracellular mediators of the vasodilator effect of exenatide we performed a series of experiments. Prior to contracting the vessels with epinephrine we preincubated the vessels (n = 5 of each experiment) with different materials. To determine whether the vasodilation due to exenatide evoked via the GLP-1R, we preincubated vessels with GLP-1R antagonist exendin(9–39) (32 μM, 30 min). Because the affinity of exendin(9–39) to bind GLP-1R is smaller than that of exenatide, we applied a ten times higher concentration of the receptor antagonist than the highest dose of exenatide [18]. In one set of experiments we mechanically removed the endothelium of the vessels by gently rubbing a hair through it. The effect of denudation was verified by the loss of response to 3 μM acetylcholine. We incubated one group of vessels with the eNOS inhibitor L-NAME (300 μM, 30 min). Other vessels were incubated with the potent heme oxygenase inhibitor Tin-protoporphyrin IX dichloride (10 μM, 30 min), others with DL-Propargylglycine, inhibitor of cystathionine-γ-lyase (10 mmol/l, 30 min), or with the relatively selective COX-1 inhibitor indomethacin (3 μM, 30 min). We tested the effects of free radical scavengers superoxide dismutase (SOD; 200 U/ml, 30 min) and catalase (1000 U/ml,30 min). H89 hydrochloride (5 μM, 30 min) was used to block protein kinase A (PKA), and 1H-(1,2,4)oxadiazolo(4,3-a)quinoxalin-1-one (ODQ, 3 μM, 30 min) was used to inhibit the effect of soluble guanylyl cyclase (sGC). To block the large-conductance calcium-activated potassium channels (BK_Ca_ channels) some vessels were incubated with tetraethylammonium (TEA, 2 mM) for 30 minutes [13]. To block the ATP-sensitive potassium (K_ATP_) channels we used glibenclamide (10 μM, 30 min) [13]. KCNQ-type voltage-dependent potassium channels were blocked by incubation with XE991 (30 μM, 15 min) [14]. The Na^+^/Ca^2+^-exchanger was blocked by incubation with its specific inhibitor SEA0400 (4 μM, 30 min) [19].

To exclude the effect of spontaneous vessel relaxation we performed untreated time-control experiments; however, the spontaneous vessel relaxation of untreated aortic rings was not significant. To test the effect of the specific inhibitors on the permanence of the epinephrine-induced plateau, we performed a row of control experiments, and found that most of the chemicals had a slight vasodilatory effect which could not have a significant influence on the results.

Chemicals were purchased from Sigma-Aldrich, St. Louis, MO, USA, except for Tin-protoporphyrin IX dichloride, which was purchased from Santa Cruz Biotechnology (Dallas, Texas, USA); XE991 was purchased from Ascent Scientific Ltd. (Avonmouth, Bristol, UK), and epinephrine was purchased from Richter-Gedeon Hungary (Budapest, Hungary). SEA0400 was synthesized in the Institute of Pharmaceutical Chemistry, University of Szeged, Szeged, Hungary by Professor Ferenc Fülöp.

Myodaq 2.01 M610+ software was used for data acquisition and display. We expressed the rate of relaxation caused by exenatide as the percentage of the contraction evoked by epinephrine.

### Statistical analysis

Statistical analysis was performed by using SPSS Version 19.0 (SPSS Inc., Chicago, IL, USA) and GraphPad Prism 6.0 (GraphPad Software Inc., La Jolla, CA, USA). Statistical significance was calculated using Student’s t-test or ANOVA with Bonferroni post hoc test as appropriate. Values are shown as mean ± SE. A value of *P* less than 0.05 was considered to be significant.

## Results

### Exenatide relaxes rat thoracic aorta in a dose-dependent manner

**Figure 1:**
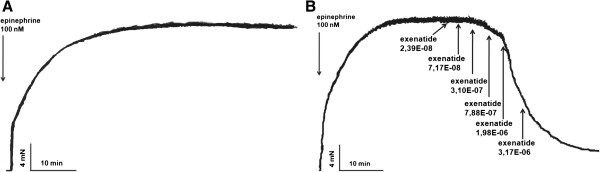
**Effect of exenatide on the vasoactivity of rat thoracic aorta.** Original records of myography experiments. Time-control of an epinephrine contracted aortic segment **(A).** Dose-dependent vasodilatory effect of exenatide on rat thoracic aorta following epinephrine contraction. 23.9, 71.7, 310, 788, 1980, 3170 nanomoles of exenatide were used to relax the vessels **(B)** (n = 5 of each experiment).

After precontracting the vessels with epinephrine, time-control experiments showed that spontaneous vessel relaxation was not significant (Figure 1A). Following the epinephrine-induced contraction, in an other set of experiments we administered increasing doses of exenatide to the organ baths to assess the vasoactive effect of this GLP-1R agonist. We found a dose-dependent relaxation of the rat thoracic aorta due to exenatide (Figure 1B).

### Role of GLP-1 receptor

**Figure 2:**
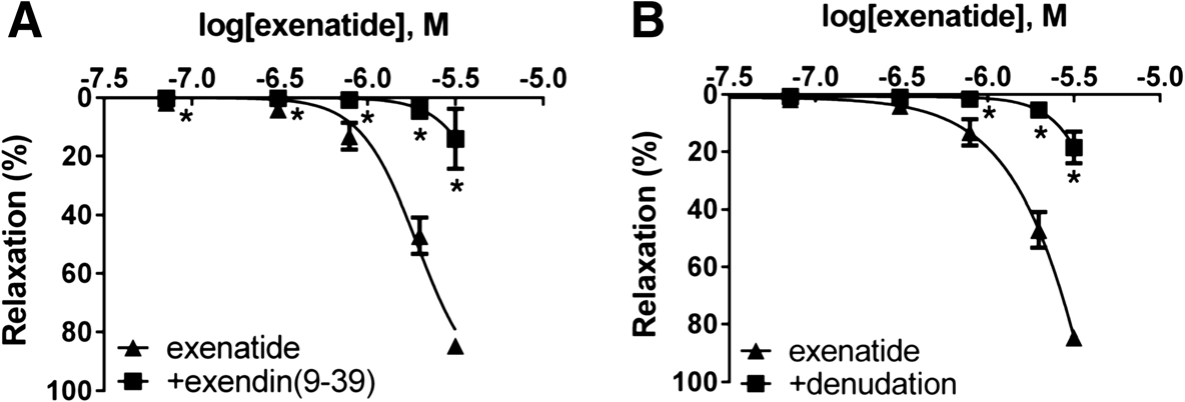
**Role of GLP-1 receptor and endothelial denudation in the vasodilatation due to exenatide.** Exenatide concentration-relaxation curves of vessels treated with exenatide only (▲) and vessels preicubated with GLP-1R anatagonist exendin(9–39) (■) **(A)**. Vasodilation evoked by exenatide in endothelium-intact and endothelium-denuded vessels **(B).** 23.9, 71.7, 310, 788, 1980, 3170 nanomoles of exenatide were used to relax the vessels (n = 5 of each experiment), *P < 0.01 compared to exenatide only (at respective concentration of exenatide).

In our experiments exenatide induced vasodilation in a GLP-1R dependent manner, since preincubation with the specfic GLP-1R antagonist exendin(9–39) almost entirely blocked the vasodilation when the maximal dose of exenatide was applied, and totaly inhibited relaxation when smaller concentrations of the GLP-1 agonist were administered to the chambers (Figure 2A).

### Effects of exenatide after endothelial denudation

When the endothelium of the thoracic aorta was mechanically removed, the relaxation due to exenatide was significantly decreased (Figure 2B).

### Effect of exenatide on the production of gasotransmitters

**Figure 3:**
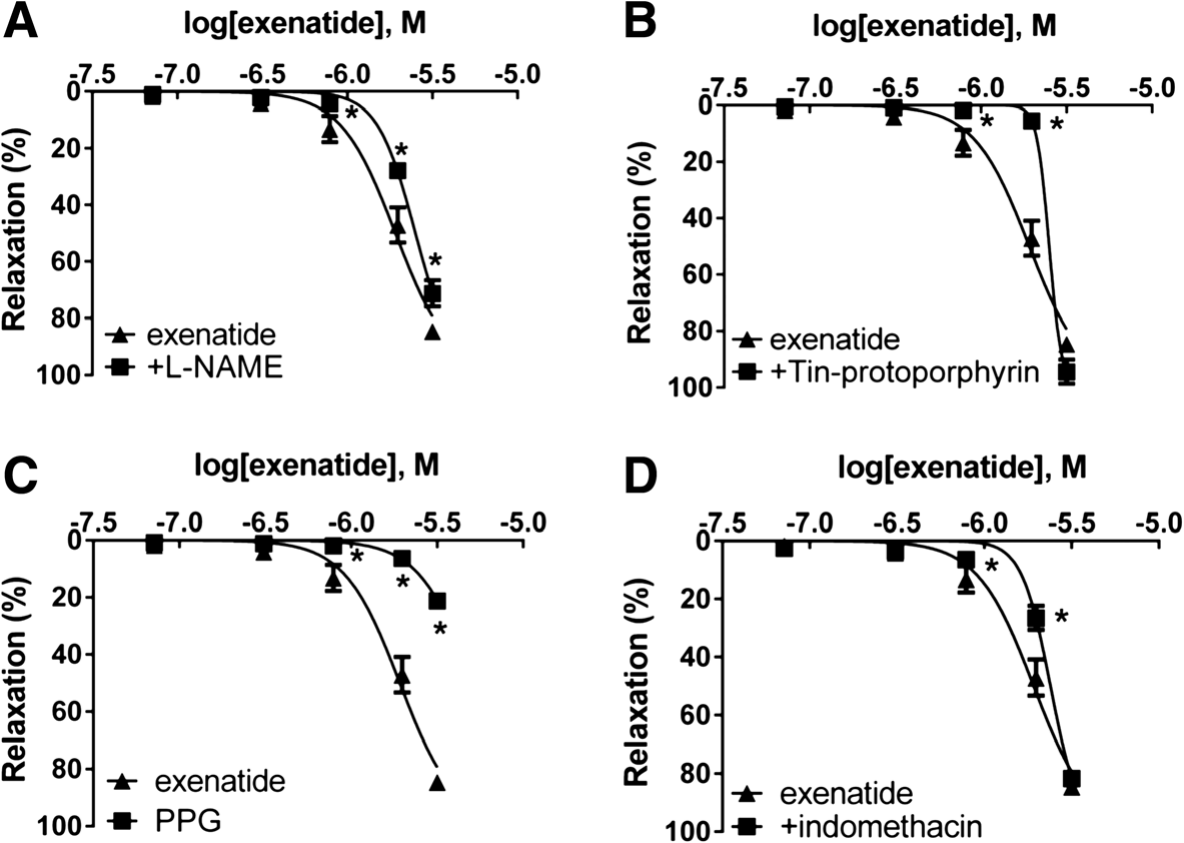
**Role of gasotransmitters and prostaglandins in the vasodilatory effect of exenatide.** Inhibition of eNOS with 300 μM *N*_ω_-Nitro-L-arginine methyl ester hydrochloride (L-NAME) **(A)**. Inhibition of CO production by blocking heme oxygenase enzyme with 10 μM Tin-protoporphyrin IX dichloride **(B)**. Blocking H_2_S production by inhibiting cystathionine-γ-lyase with 10 mM DL-Propargylglycine (PPG) **(C)**. Inhibition of prostaglandin production with 3 μM indomethacin **(D)**. 23.9, 71.7, 310, 788, 1980, 3170 nanomoles of exenatide were used to relax the vessels (n = 5 of each experiment), *P < 0.01 compared to the relaxation caused by exenatide only (at respective concentration of exenatide).

Incubation of vessels with the eNOS inhibitor L-NAME led to a mild but significant decrease in the relaxation of the rat thoracic aorta (Figure 3A). To determine further mediators of the vasodilator effect of exenatide, we examined the role of CO and H_2_S. When we preincubated vessels with Tin-protoporphyrin, a potent heme oxygenase inhibitor, the vasorelaxation to exenatide was significantly reduced (Figure 3B). The inhibition of NO-synthesis and the inhibiton CO-production only partially decreased the rate of vasodilation: we therefore wished to prove that the third gasotransmitter, H_2_S also plays a part in the vasoactive effect of exenatide. The inhibition of cystathionine-γ-lyase by preincubating vessels with PPG resulted in a significant decrease in the rate of relaxation (Figure 3C). Comparing the effects of these three gasotransmitters leading to vasodilation in response to exenatide, H_2_S seemed to have the most remarkable effect.

### Effect of the inhibition of prostaglandin biosynthesis

Incubation of vessels with the COX inhibitor indomethacin for 30 minutes significantly decreased vasodilation to exenatide (Figure 3D).

### Free radicals play a part in the vasoactive effect of exenatide

**Figure 4:**
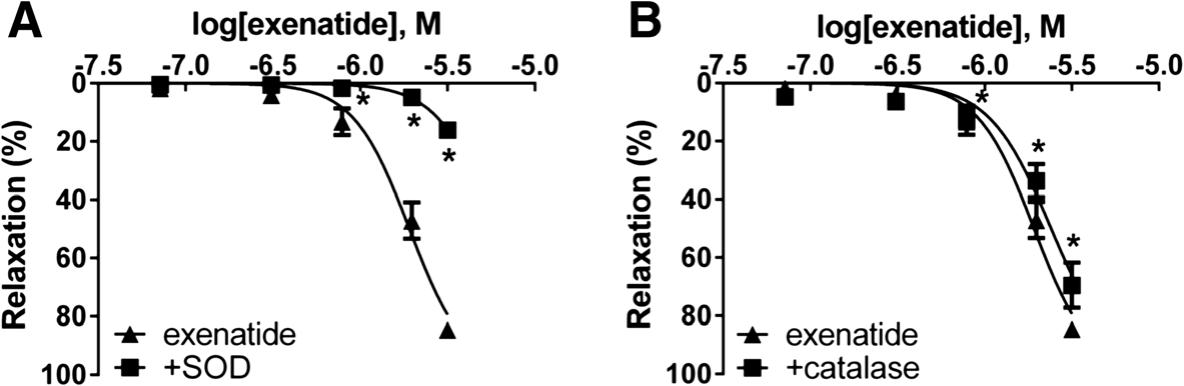
**Effect of free radicals on the vasodilation due to exenatide.** Concentration-relaxation curve of exenatide with/without the addition of 200 U/ml of the free radical scavanger superoxide dismutase (SOD) **(A)**. Concentration-relaxation curve analyzing the possible role of hydrogen peroxide by blocking its formation with 1000 U/ml catalase **(B)**. 23.9, 71.7, 310, 788, 1980, 3170 nanomoles of exenatide were used to relax the vessels (n = 5 of each experiment), *P < 0.01 compared to the relaxation caused by exenatide only (at respective concentration of exenatide).

To determine the role of ROS in the vasorelaxation caused by exenatide, we preincubated vessels with superoxide dismutase or with catalase. The rate of relaxation was significantly decreased in both experiments; however, SOD proved to be a more potent inhibitor of vasodilation to exenatide (Figure 4).

### Effects of inhibiting the cAMP-dependent protein kinase A and cGMP-dependent protein kinase G

**Figure 5:**
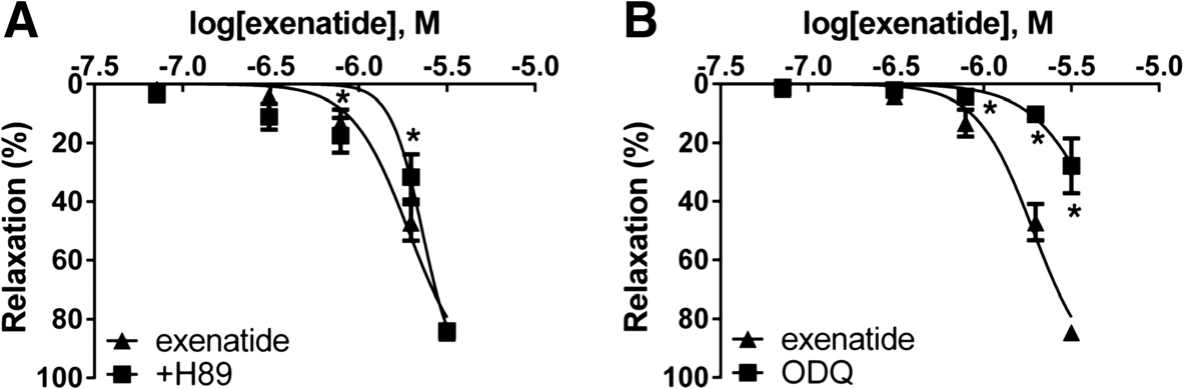
**Concentration-relaxation curves showing the possible effector molecules of the exenatide induced vasodilation.** Blocking cAMP-dependent protein kinase A (PKA) with 5 μM H89 hydrochloride **(A)**. Inhibiton of soluble guanylyl cyclase with 3 μM 1H- (1,2,4) (ODQ) **(B)**. 23.9, 71.7, 310, 788, 1980, 3170 nanomoles of exenatide were used to relax the vessels (n = 5 of each experiment), *P < 0.01 compared to the relaxation caused by exenatide only (at respective concentration of exenatide)

In order to determine the second messenger of the dilatation caused by exenatide, we incubated vessels with H89, an inhibitor of PKA. This caused only a mild decrease in the vasorelaxation at a low concentration (Figure 5A). In turn, inhibition of soluble guanylyl cyclase by ODQ significantly inhibited the vasorelaxation at higher concentrations as well (Figure 5B).

### Role of potassium channels in the vasodilator effect of exenatide

**Figure 6:**
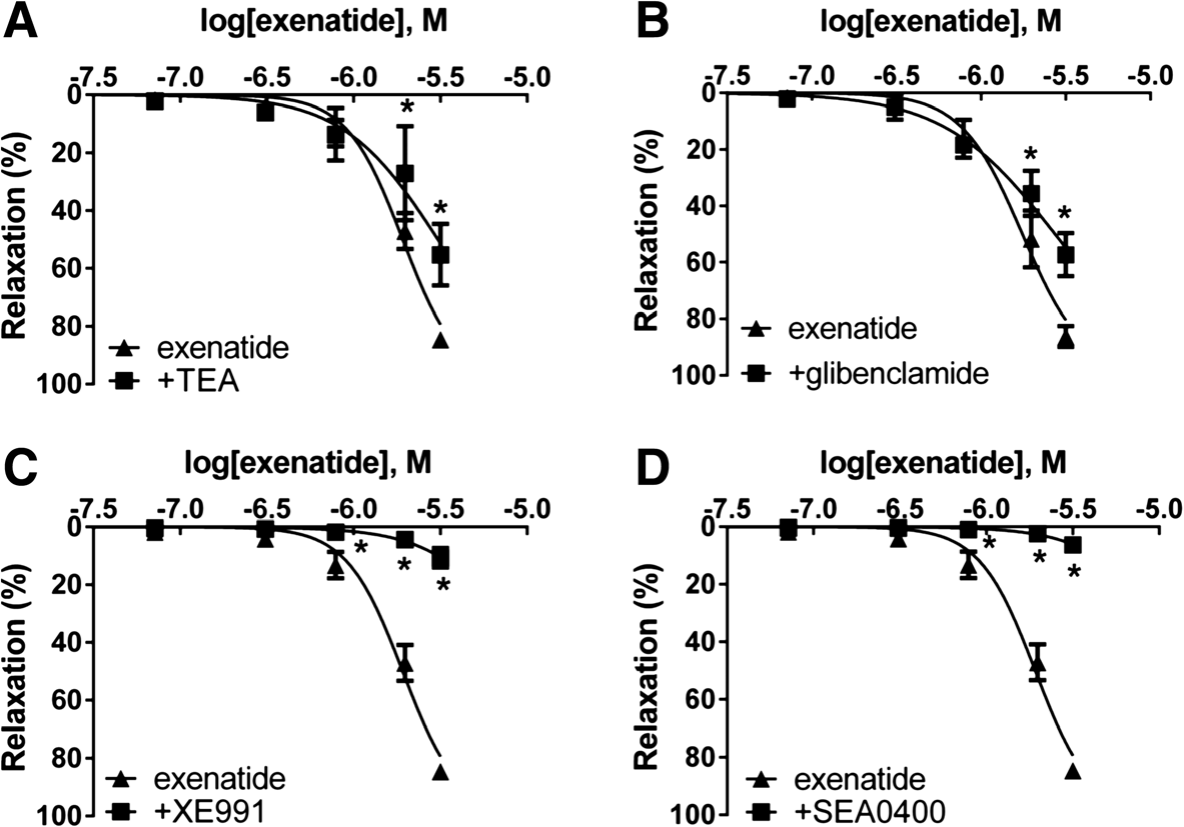
**Role of potassium channels and the Na**^**+**^**/Ca**^**2+**^**-exchanger in the vasodilator effect of exenatide.** Blockade of large-conductance calcium-activated potassium channels with 2 mM tetraethylammonium (TEA) **(A)**. Inhibition of ATP-sensitive potassium channels with 10 μM glibenclamide **(B)**. KCNQ-type K_v_ channels blocked by 30 μM XE991 **(C)**. Selective inhibition of the Na^+^/Ca^2+^-exchanger with 4 μM SEA0400 **(D)**. 23.9, 71.7, 310, 788, 1980, 3170 nanomoles of exenatide were used to relax the vessels (n = 5 of each experiment), *P < 0.01 compared to the relaxation caused by exenatide only (at respective concentration of exenatide).

Preincubating vessels with three different potassium channel blockers before adding increasing concentrations of exenatide to the myograph chambers resulted in a signifcant decrease in the relaxation of all cases. Incubating one group of vessels with TEA, an inhibitor of the BK_Ca_ channels, demonstrated inhibition of vasodilation (Figure 6A). Relaxation was also inhibited by a blockade of the K_ATP_ channels by preincubation with glibenclamide (Figure 6B). In the group of vessels preincubated with XE991, a KCNQ (a type of K_v_ channels) channel inhibitor, most of the vasodilation was abolished (Figure 6C). However, there was a less pronounced decrease of vasodilation with the inhibition of BK_Ca_ and K_ATP_ channels compared to the inhibition of K_v_ channels.

### Effects of inhibiting the Na^+^/Ca^2+^-exchanger with SEA0400

We found that SEA0400, an inhibitor of the Na^+^/Ca^2+^-exchanger markedly inhibited vasorelaxation. Preincubation of vessels with SEA0400 almost completely abolished the whole of the vasodilation caused by exenatide (Figure 6D).

## Discussion

**Figure 7:**
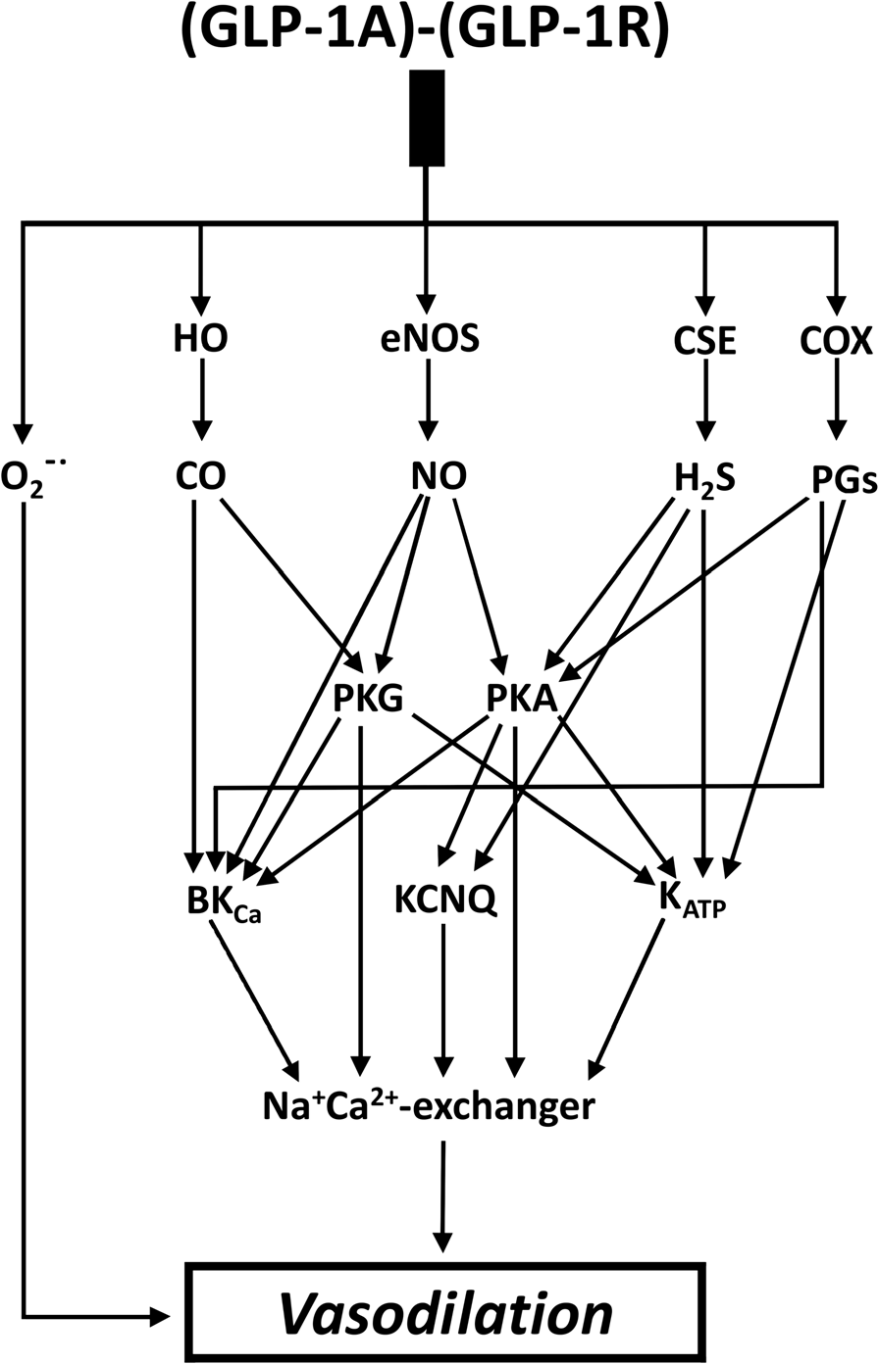
**Hypothetical mechanism of the exenatide induced vasodilation according to our data and according to previous examinations [**[3]**-**[7]**,**[10]**-**[14]**,**[19]**-**[31]**].** HO: heme oxygenase, eNOS endothelial nitric oxide synthase, CSE: cystathionine-γ-lyase, COX: cyclooxygenase, PG: prostaglandin, H_2_S: hydrogen sulphide NO: nitric oxide, CO: carbon monoxide, O_2_^–•^:superoxide anion, PKA: cAMP-dependent protein kinase, PKG: cGMP-dependent protein kinase, BK_Ca_: large-conductance calcium activated potassium channel, KCNQ: a type of voltage-gated potassium channel, K_ATP_: ATP-sensitive potassium channel.

In our study we demonstrated that exenatide causes vasorelaxation of isolated rat thoracic aorta in a dose-dependent manner. Based on our findings, the hypothetical mechanism of the vasorelaxation caused by exenatide is as follows: exenatide binds to GLP-1R and activates both endothelial and vascular smooth muscle cells, leading to the production of H_2_S, NO, CO, O_2_^–•^, H_2_O_2_ and prostaglandins. Formation of these relaxing factors contributes to the activation of potassium channels either directly or by activating PKG or - to a lesser extent - PKA. Subsequent activativation of the Na^+^/Ca^2+^-exchanger resulting in calcium efflux leads to smooth muscle relaxation, and thus vasorelaxation (Figure 7).

While GLP-1 amides were found to have GLP-1R-dependent and –independent vasodilatory effects, in our experiments exenatide caused relaxation of the rat thoracic aorta in a GLP-1R dependent manner [1]. This finding is congruent with a study that demonstrated that GLP-1R agonists reduce systolic blood pressure via the GLP-1R, however the same study reported that the antihypertensive effect was NO-independent and that vasodilaton is evoked by the increased secretion of the atrial natriuretic peptide (ANP) due to GLP-1R activation [32]. Non-PKA GLP-1R-dependent effects have been shown in the regulation of eNOS expression in aortic endothelial cells in an ApoE^-/-^ mouse model, suggesting the role of GLP-1 agonists in the inhibition of endothelial cell dysfunction [33].

Previously exendin-4 was found to relax isolated rat thoracic aorta involving K_ATP_ and cAMP, although the relaxation caused by endogenous GLP-1 amides was greater than those caused by the synthetic peptides [3]. The same study stated that the relaxation caused by physiological isoform GLP-1(7–36)amide was not altered by endothelial denudation or incubation either with L-NAME, indomethacin or catalase [3]. Conversely, we found that these vasodilators are only partially responsible for the relaxation caused by exenatide, indicating a role of the endothelium in vasorelaxation due to exanatide but not the endogenous GLP-1-amides [3].

Endogenous GLP-1 has been shown to cause endothelium-dependent vasorelaxation via NO; however, data regarding GLP-1-induced endothelium-independent vasorelaxation have also been published [4-7]. NO, formed by the endothelial nitric oxide synthase (eNOS) enzyme, acts via activating soluble guanylyl cyclase (sGC) leading to an increased production of cyclic-guanosine monophosphate (cGMP) which activates the cGMP-dependent protein kinase (PKG) [10]. Alternatively, NO was shown to increase cAMP levels in cardiac myocytes by activating adenylyl cyclase in a cGMP*-*independent manner, resulting in the activation of the cAMP-dependent protein kinase (PKA) [20]. On the other hand, in vascular smooth muscle cells NO may have vasodilatory effect via the direct activation of the large-conductance calcium activated potassium channels (BK_Ca_ channels) [13]. In our experiments we demonstrated that NO is only partially responsible for the vasodilator effect of exenatide in rat thoracic aorta. This finding is in contrast with a previous study where endothelial denudation and preincubation with L-NAME had no significant effect on the vasodilation caused by endogenous GLP-1 in rat thoracic aorta [3]. Although GLP-1 was shown to cause significant vasorelaxation of rat saphenous artery and recruitment of mucle microvasculature via the NO/PKA pathway, we found that these mechanism are only partially responsible for the vasodilation evoked by exenatide in the thoracic aorta of rats [34]. The possible explanation for the discrepancies between former studies and ours may be that we studied the effects of the GLP-1 agonist exenatide while in previous studies the endogenous, physiological isoform GLP-1(7–36) amide was used [3-6].

Although previously only NO was found to be involved in the GLP-1-induced vasodilatation [4-7] among the three gasotransmitters, we found that CO and H_2_S also contribute. CO, an important regulator of vascular tone, is a vasodilator gaseous molecule formed from heme by heme oxygenase (HO) in vascular smooth muscle cells [10]. Similar to NOS, HO has three isoforms, of which HO-2 is constitutively expressed in endothelial cells and vascular smooth muscle cells [10,21]. CO acts through the activation of sGC, but also increases the calcium sensitivity of BK_Ca_ channels, which in turn leads to smooth muscle hyperpolarization [22]. CO may also cause vasodilation through the activation of voltage-dependent potassium channels (K_v_) [22].

We also demonstrated the involvement of the third gasotransmitter, H_2_S, in mediating the vasodilatory effect of exenatide. H_2_S is formed in endothelial cells and vascular smooth muscle cells from homocystein, cysthationine or L-cysteine by cystathionine-γ-lyase, cystathionine-β-synthase and 3-mercaptopyruvate-sulfurtransferase (3MST) [23]. H_2_S has been shown to act via the sGC-cGMP-PKG pathway and also via to the activation of ATP-sensitive and KCNQ-type voltage-gated potassium channels (K_ATP_), causing hyperpolarization of smooth muscle cells [11,24,35]. H_2_S was also shown to activate adenylyl cyclase, which generates cAMP, thereby activates PKA leading to vasodilation [25].

In contrast with previous findings, our experiments showed that prostaglandins and reactive oxygen species are also involved in the vasorelaxation due to exenatide, although to a variable extent. In certain arteries reactive oxygen species (ROS) play a role in the mediation of vasodilation [36]. They were also found to relax both pulmonary and mesenteric arteries [37,38]. In superoxide-dismutase (SOD) knockout mice eNOS-dependent endothelium-mediated vasorelaxation is impaired, suggesting the role of superoxide anion (O_2_^–•^) in vasodilation [39].

Potassium channels are often targets of gaseous mediators, resulting in vasodilation [13]. K_ATP_ and K_v_ channels are inhibited by GLP-1 in β-cells [8,9], but we found that both channels are activated by exenatide in the vasculature, leading to vasodilation of the isolated rat thoracic aorta. Moreover, in our experiments we demonstrated that the blockade of KCNQ type voltage-gated potassium channels abolished the vasodilation caused by exenatide, which has not been observed before. GLP-1 was formerly shown to cause vasodilation of rat thoracic aorta via the activation of K_ATP_ channels [3]. In a human ischaemia-reperfusion injury model subcutaneously administered exenatide was found to be protective against endothelial-dysfunction via the opening of K_ATP_ channels [40]. We found that inhibition of K_ATP_ channels significantly decreased the exenatide evoked vasodilation, altough it did not appear to be as impressive as the inhibition of KCNQ type K_v_ channels.

Activation of BK_Ca_ channels by exenatide has not been described previously. Nevertheless, we found that the blockade of these channels with TEA also inhibited vasorelaxation, but that this occurred only in higher concentration. It is well-established that NO and CO exert vasodilatory effects by activating BK_Ca_ channels [10,13].

We demonstrated that the inhibition of the sodium-calcium exchanger (Na^+^/Ca^2+^-exchanger or NCX) markedly inhibited vasodilation evoked by exenatide, which has not been shown before. NCX is a transmembrane protein located in almost all cell types [26]. The key function of NCX is to deliver calcium with a simultaneous entry of sodium into the cell upon repolarization, whereas when the cell membrane is depolarized, its activity is reversed and it induces calcium influx [26,27]. NO was observed to decrease the vasoconstriction mediated by the NCX in rat aorta [28].

In this study we provide *in vitro* evidence for the possibility that GLP-1 receptor agonist exenatide decreases central (aortic) blood pressure. Central blood pressure indicates the load affecting the left ventricle, the coronary and cerebral vessels, and it correlates closely with the risk of cardiovascular (CV) events, thereby demonstrating the value of our findings in the clinical context [41]. The CAFE study established that central aortic pressure is a strong predictor of clinical outcomes [42]. There is also *in vivo* evidence that exenatide reduces both systolic and diastolic blood pressure [43]. Moreover, GLP-1 receptor agonists are associated with outstanding improvements of other CV risk factors such as body weight and lipid profiles, while they have only a small effect on heart rate and QT_c_. [44]. Exenatide was found to be more beneficial than biguanides, dipeptidyl peptidase-4 inhibitors, thiazolidindiones, or basal insulin, in reaching the therapeutic goals recommended by the American Diabetes Association (ADA) in the treatment of type 2 diabetes, which is also promising in the reduction of cardivascular risk [45]. Treatment with exenatide has shown to have similarly beneficial effects on microvascular endothelial function, oxidative stress, vascular activation and markers of inflammation as metformin in patients pre-diabetes and obesity [46]. Taking the above mentioned into consideration, the ability of a drug used for the treatment of diabetes to further lower central (aortic) blood pressure may be highly beneficial.

Our study and relevance of our findings are limited in the use of single methodolgy (myography) and the specificity of the inhibitors.

## Conclusions

In conclusion, we demonstrated that exenatide may lower central (aortic) blood pressure by in a GLP-1R-dependent manner mainly via H_2_S but also via NO, CO, O_2_^–•^ and prostaglandins, and that this effect can be mediated via the activion of PKA and PKG. Through the induction of these mediators, exenatide also influences the activity of potassium channels and the Na^+^/Ca^2+^ -exchanger.

## Abbreviations

BKCa: Large-conductance calcium activated potassium channel; CO: Carbon monoxide; CV: Cardiovascular; GLP-1: Glucagon-like-peptide-1; GLP-1R: Glucagon-Like-Peptide-1 receptor; NO: Nitric oxide; HO: Heme oxygenase; H2S: Hydrogen sulphide; H89: N-(2-(p-Bromocinnamylamino)ethyl)-5-isoquinolinesulfonamide dihydrochloride; KATP: ATP-sensitive potassium channel; Kv: Voltage-gated potassium channel; L-NAME: *N*_ω_-Nitro-L-arginine methyl ester hydrochloride; 3MST: 3-mercaptopyruvate-sulfurtransferase; NOS: Nitric oxide synthase; ODQ: 1H-(1,2,4)oxadiazolo(4,3-a)quinoxalin-1-one; PKA: Protein kinase A; PKG: Protein kinase G; PPG: DL-Propargylglycine; ROS: Reactive oxygen species; sGC: Soluble guanyly cyclase; SOD: Superoxide dismutase; TEA: Tetraethylammonium chloride.

## Competing interests

The authors declare that they have no competing interests.

## Authors’ contributions

The experiments were designed by IW, GAM. and ES. Experiments were performed by ES. Data analysis was carried out by ES, GAM, SZK, IASZ, BL and IW. Financial and material support was provided by IW and FF. The paper was written by ES, SZK, IW and GAM. Discussion and manuscript review are contributed by BL, TK, FF, GAM and IW. All authors read and approved the final manuscript.
